# Regulation of Cerebral BDNF, VEGF, and GluN2B Gene Expression and Cytokine Levels by Riparin A in a Murine Model of Depression

**DOI:** 10.1155/adpp/6965826

**Published:** 2025-08-30

**Authors:** Raphaela Gonçalves Barros, Julia Nunes Estrela de Carvalho, Cássio Prinholato da Silva, Felipe Garcia Nishimura, Rene Oliveira Beleboni

**Affiliations:** ^1^ Department of Biotechnology, University of Ribeirão Preto, Ribeirão Preto, Brazil, unaerp.br; ^2^ Department of Pathology and Immunology, Baylor College of Medicine, Texas Children’s Hospital, Houston, Texas, USA, texaschildrens.org; ^3^ Texas Children’s Microbiome Center, Texas Children’s Hospital, Houston, Texas, USA, texaschildrens.org; ^4^ Department of Pathology, Texas Children’s Hospital, Houston, Texas, USA, texaschildrens.org; ^5^ Laboratory of Structural Biology and Protein Engineering, Carlos Chagas Institute, FIOCRUZ Paraná Curitiba, Paraná, Brazil, icc.fiocruz.br

**Keywords:** antidepressant, CUMS mode, cytokines, interleukins, Riparins, tyraminic compounds

## Abstract

Riparin A is a synthetic compound with established antidepressant and anxiolytic properties. Given its therapeutic potential and the crucial roles of brain‐derived neurotrophic factor (BDNF), vascular endothelial growth factor (VEGF), and the GluN2B subunit of the N‐methyl‐D‐aspartate (NMDA) receptor in the pathophysiology and treatment of depression, this study aimed to evaluate the effects of Riparin A on the expression of these neurotrophic factors and receptor subunit in the hippocampus and cortex of rats subjected to the chronic unpredictable mild stress (CUMS) model of depression. Using RT‐qPCR, we observed that Riparin A significantly upregulated BDNF and VEGF mRNA levels while downregulating GluN2B expression, remarkably on the hippocampal area. Furthermore, ELISA assays revealed that Riparin A modulated neuroinflammation by reducing proinflammatory cytokines TNF‐α and IL‐1β while increasing anti‐inflammatory cytokines IL‐4 and IL‐10. These findings support the antidepressant properties of Riparin A and shed light on its underlying mechanisms, reinforcing the interplay between neurotrophic and inflammatory pathways in pathophysiology of depression and its treatment.

## 1. Introduction

Major depression (MD) is an important psychiatric disorder with chronic and recurrent nature, affecting over 300 million people worldwide and causing significant social and economic impacts [1]. Despite several therapeutic approaches and medications for depression, 10%–30% of patients do not show significant symptom relief through the treatment. Additionally, side effects such as motor disturbances, cognitive dysfunction, libido impairment, and changes in weight and sleep can easily occur with conventional treatment, adversely affecting the therapeutic adherence [2]. This underscores the need for more effective and safer antidepressant drugs, as well as further biochemical studies for a better understanding of neuromolecular bases of depression.

The exact etiology and pathogenesis of depression remain unclear; however, recent research has shown that the inflammatory response may be involved, with interleukins such as IL‐1β and IL‐10 being strongly implicated, along with the modulation of genes BDNF, VEGF, and GluN2B in the hippocampus and cortex of animal models of depression [3, 4]. Importantly, postmortem studies have revealed decreased levels of mature BDNF and increased expression of its precursor proBDNF and the BDNF propeptide in patients with MD, suggesting a shift in the balance of neurotrophic support that may contribute to disease pathology [5]. Additionally, reduced BDNF transcript variants have been reported in the hippocampus and dorsolateral prefrontal cortex (DLPFC) of individuals with MD, further indicating region‐specific dysregulation of BDNF signaling [6–8]. Alterations in VEGF expression have also been observed in the hippocampus of depressed individuals, potentially impacting neurogenesis and vascular support [9, 10]. Furthermore, GluN2B, a subunit of the NMDA receptor implicated in synaptic plasticity, has been shown to be dysregulated in depression, affecting excitatory neurotransmission and neuronal remodeling [9, 11].

Neurotrophins, individually or collectively, exhibit important roles on the normal brain dynamics, acting in neuronal remodeling and plasticity, in the development, maturation, and differentiation of new neurons, which is crucial in different brain functions, including cognition and mood regulation [12, 13].

In addition to these neurotrophic factors, some cytokines have been highlighted within the clinical picture of depression, with some being studied as potential biomarkers for this disorder, such as IL‐1β and IL‐10 [3, 14, 15]. Cytokines are involved in many aspects related to the pathophysiology of depression, including neurotransmitter metabolism, neuroendocrine function, neurogenesis, neuronal integrity, synaptic remodeling, and neural plasticity.

Among new preclinical alternatives for the treatment of depression, Riparins, a class of tyramine compounds with antidepressant and anxiolytic properties, have become a promising pharmacological option. Riparins are found in several plant species, primarily those of the genus *Aniba* [16, 17]. The species *Aniba riparia* (Nees) Mez presents three different compounds with promising preclinical activities: Riparins I and III, which have antidepressant and anxiolytic activities, and Riparin II, which presents anxiolytic effects only [18–21]. Recently, we demonstrated the anxiolytic and antidepressant activity of Riparin A, a synthetic compound similar to the other Riparins, in animal models under acute treatment [22] and observed in a chronic depression model that Riparin A reduces the duration of immobility and attenuates anhedonia in animals [23]. Given these results and the relevance of better understanding the mechanism of action of Riparin A, this study aimed to evaluate the effects of Riparin A on BNDF, VEGF and GluN2B genes expression and cytokine modulation in different brain areas in animals subjected to the chronic unpredictable mild stress (CUMS) model of depression.

## 2. Materials and Methods

### 2.1. Drugs

Riparin A [N‐(2‐phenylethyl)benzamide] was purchased from Toronto Research Chemicals (TRC, Toronto, Canada) (Cat# P296050–Lot 2‐KAA‐67‐1). Fluoxetine hydrochloride (FLX) was obtained from Sigma‐Pharma (Brazil).

### 2.2. Animals

This study received approval from the Ethics Committee for Research at the University of Ribeirão Preto under protocol number: 09/2015. All experimental protocols involving animals were established according to the guidelines for the use of animals in research as per the Brazilian College of Animal Experimentation and the American Guidelines for Animal Care, ensuring the humane treatment of animals. The animals were acquired from ANILAB (Paulínia, SP) and housed in our local animal facility. Male Wistar rats (150–200 g) were used and housed in groups of five animals per cage and maintained under a controlled 12/12‐h light/dark cycle, with a temperature of 20 ± 2°C and humidity at 55%. They had access to food and water ad libitum (with some days during the CUMS protocol involving water and food deprivation).

Prior to euthanasia, animals were anesthetized with an intraperitoneal injection of a combination of xylazine (2%) and ketamine (10%) to ensure adequate sedation.

Following decapitation, the brains were promptly removed and placed on aluminum foil in proximity to an ice‐cooled Petri dish to facilitate dissection of the hippocampus and cerebral cortices from each animal. Upon completion of the dissections, the tissues were transferred to appropriately labeled eppendorf tubes, weighed, and stored at −70°C until further analysis.

### 2.3. CUMS Procedure and Experimental Design

The CUMS procedure was performed following Kumar et al.’s study [24] with minor modifications. Animals were randomly allocated into four groups (*n* = 7 per group): a nonstressed saline control (CN), a stressed group receiving vehicle (CUMS), a CUMS group treated with Riparin A (10 mg/kg; CUMS/RIP), and a CUMS group treated with fluoxetine hydrochloride (CUMS/FLX).

Throughout the 29‐day CUMS protocol, animals were exposed to a different mild stressor each day in a semirandomized, unpredictable order to avoid habituation (Table 1). Treatments were administered once daily via intraperitoneal (i.p.) injection for 21 consecutive days, beginning on Day 7 and continuing through Day 29, always 30 min before the stress exposure or any experimental procedure. The dose of Riparin A (10 mg/kg) was selected based on previously obtained antidepressant results [22]. Fluoxetine (10 mg/kg) was administered intraperitoneally 30 min before each experiment, when applicable.

**Table 1:** Schedule of the chronic unpredictable mild stress procedure.

Week	Monday	Tuesday	Wednesday	Thursday	Friday	Saturday	Sunday
Week 1	Cold swim	Food/water deprivation	24 h light	Normal swim	Food/water deprivation	Dirty cage	No stress
Week 2	Inclined cage	Cold swim	Food/water deprivation	24 h light	Normal swim	No stress	Dirty cage
Week 3	Food/water deprivation	Normal swim	Inclined cage	No stress	24 h light	Cold swim	No stress
Week 4	24 h light	Normal swim	Dirty cage	Inclined cage	No stress	Cold swim	Food/water deprivation

The stress procedures included cold swim at 12°C, 5 min, food and water deprivation for 24 h, continuous light for 24 h, normal swim at 23 ± 2°C for 15 min, dirty cage for 24 h, and inclined cage at 45° for 7 h. All stress procedures were conducted between 09:00 and 14:00 h.

### 2.4. Real‐Time PCR

RNA was isolated and purified from individual samples of the hippocampus and cortex using TRI Reagent (Sigma‐Aldrich, St. Louis, Missouri, USA) according to the manufacturer’s instructions. The concentration of extracted RNA was determined by UV spectrophotometry (260 nm), and its integrity was verified by denaturing 1% agarose gel electrophoresis under constant voltage conditions (80 V) for 60 min. cDNA was synthesized using the high‐capacity cDNA reverse transcription kit (ThermoFisher) in a total volume of 20 μL containing 1 μg of total RNA. Real‐time PCR was conducted in a final volume of 25 μL containing 1 μL of cDNA (SYBR Green JumpStart Taq ReadyMix for RT‐qPCR, Cat# S4438, Sigma‐Aldrich, USA). The amplification reactions were performed in triplicate using an MXPRO 3005 thermal cycler (Stratagene) with the following cycling conditions: 94°C for 2 min, followed by 40 cycles of 94°C for 15 s and 60°C for 1 min. The following primer sets were used: BDNF—Fwd: 5′‐GCA​GCC​TTC​TTT​TGT​GTA​ACC‐3′, Rev: 5′‐AGA​GTG​ATG​ACC​ATC​CTT​TTC‐3′ [25]; VEGF ‐ Fwd: 5′‐GGT​AGC​TGA​GGA​CGC​AGT​GT‐3′, Rev: 5′‐GGT​AGC​TGA​GGA​CGC​AGT​GT‐3′ [26]; GluN2B ‐ Fwd: 5′‐CUC​AGA​AGA​AGA​AUC​GGA​A‐3′, Rev: 5′‐UUC​CGA​UUC​UUC​UUC​UGA​G‐3′ [27]. RT‐qPCR data were analyzed according to the 2^−ΔΔCt^ method proposed by Livak and Schmittgen [28]. According to this model, the following equations were applied:

(1) f fold−change=2−∆∆Ct,∆Ct=Target gene Ct−Reference gene Ct,∆∆Ct=∆Ct12−∆Ct,∆Ct1=sample of interest,∆Ct2=control group sample.

### 2.5. Enzyme‐Linked Immunosorbent Assay (ELISA) for Cytokine Measurement

The hippocampi and cortices from animals in the different control and experimental groups described above were homogenized in a RIPA buffer supplemented with 1% protease inhibitor cocktail (Sigma‐Aldrich, St. Louis, MO, USA) on ice. The levels of TNF‐α (cat. RAB0480), IL‐1β (cat. RAB0278), IL‐4 (cat. RAB0302), IL‐6 (cat. RAB0312), IL‐10 (cat. RAB0247), and IL‐13 (cat. RAB0258) were assessed using ELISA kits (Sigma‐Aldrich). Following the manufacturer’s instructions, 100 μL of samples were pipetted into an ELISA plate precoated with antibodies against TNF‐α, IL‐1β, IL‐4, IL‐6, IL‐10, and IL‐13 and incubated for 2.5 h at 25°C with gentle agitation. The wells were then washed with wash buffer, and biotinylated antibodies against rat TNF‐α, IL‐1β, IL‐4, IL‐6, IL‐10, and IL‐13 were added to each well. After washing away unbound biotinylated antibodies, 100 μL of streptavidin‐HRP conjugate solution was added to the wells and incubated for 45 min at room temperature. All wells were washed again, and 100 μL of TMB substrate solution (chromogenic tetramethylbenzidine solution) was added and incubated for 30 min. Finally, a stop solution (2N H2SO4) was added, causing a change from blue to yellow. The optical density of each well was measured using a microplate reader at a wavelength of 450 nm in triplicate.

### 2.6. Statistical Analysis

Data were analyzed using one‐way ANOVA followed by Tukey’s post hoc test. Statistical analyses and graphical constructions were performed using GraphPad Prism 8.0 (USA), with a *p* value of < 0.05 considered statistically significant.

## 3. Results

### 3.1. Posttranscriptional Effects of Riparin A on BDNF, VEGF, and GluN2B Genes

In the hippocampus, stress had a strong effect on BDNF and VEGF gene expression, significantly repressing both (Figures 1(a) and 1(c)). Conversely, stress increased GluN2B expression. Both treatments, fluoxetine and Riparin A, significantly counteracted these effects in the stress group by increasing BDNF and VEGF expression and reducing GluN2B expression.

Figure 1Effects of CUMS and treatments on BDNF, VEGF, and GluN2B gene expression in the hippocampus and cortex. Gene expression levels of BDNF in the hippocampus and cortex (a, b), VEGF (c, d), and GluN2B (e, f) were assessed by RT‐qPCR of animals subjected to saline as control (CN), chronic unpredictable mild stress (CUMS), and CUMS treated with fluoxetine (CUMS/FLX) or Riparin A (CUMS/RIP). Statistical analysis was performed using one‐way ANOVA followed by Tukey’s post hoc test. ^∗^ *p* < 0.05, ^∗∗^ *p* < 0.005, and ^∗∗∗∗^ *p* < 0.0001.

**Figure:**
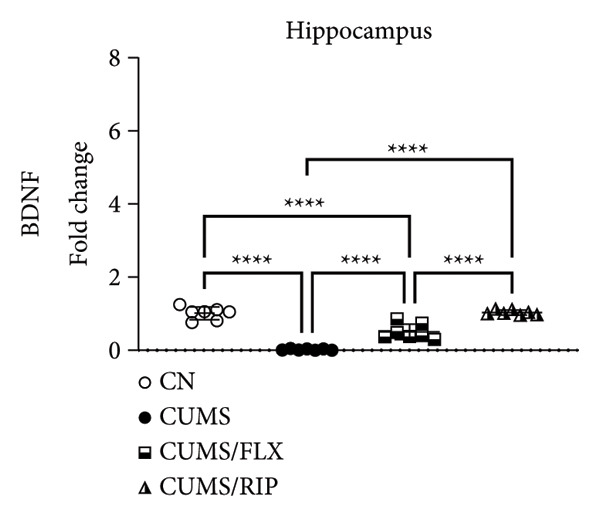
(a)

**Figure:**
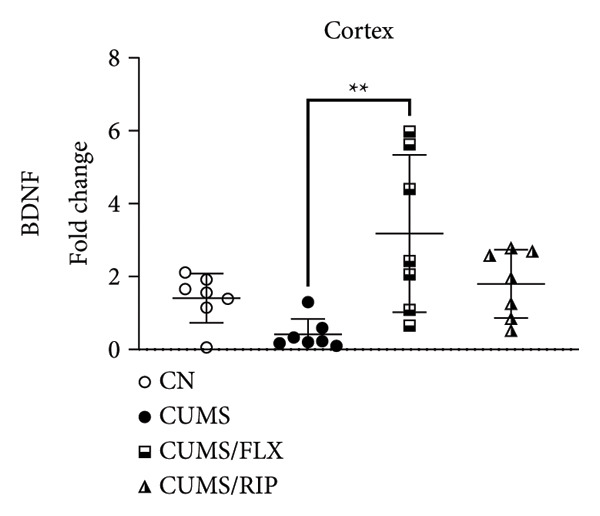
(b)

**Figure:**
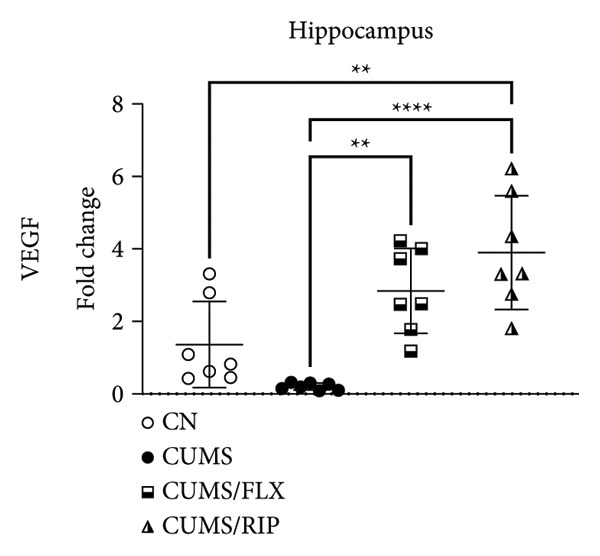
(c)

**Figure:**
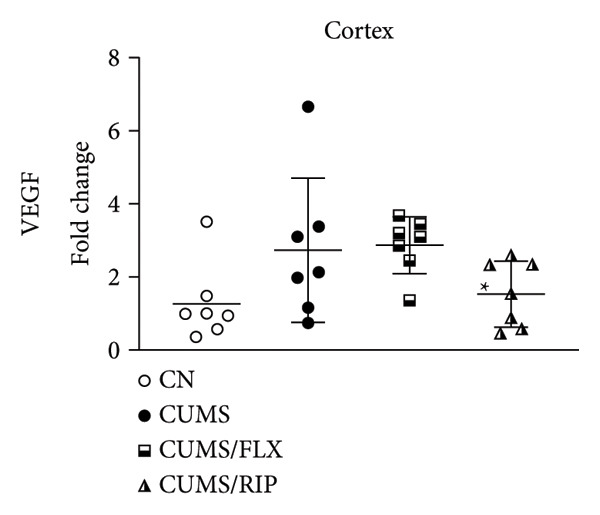
(d)

**Figure:**
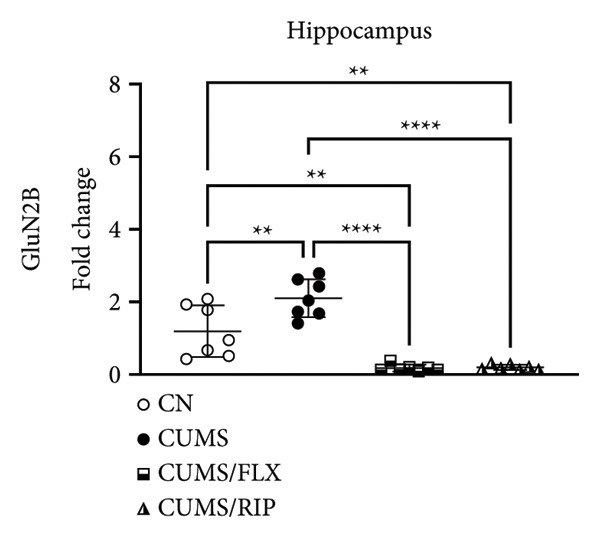
(e)

**Figure:**
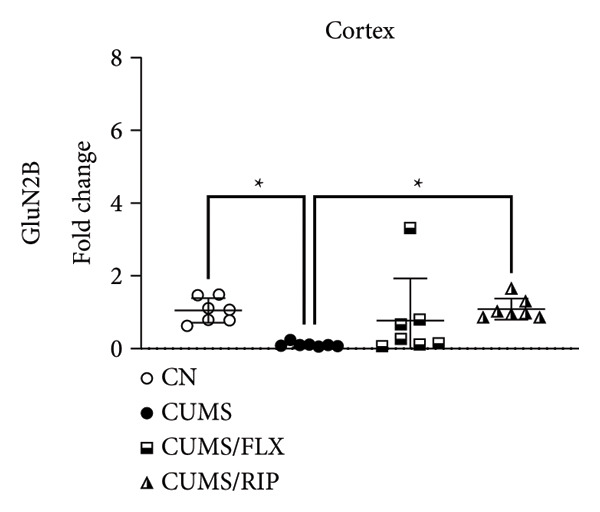
(f)

Although fluoxetine increased BDNF expression after stress, it did not fully restore it to the normal levels observed in the control group. In contrast, Riparin A successfully restored BDNF levels to baseline. Additionally, no statistical difference was observed in VEGF expression between the control and CUMS groups (Figure 1(c)). However, both fluoxetine and Riparin A increased VEGF expression after stress, with Riparin A inducing it even more than in the control group. Regarding GluN2B, both treatments significantly reduced its expression after stress, lowering it to levels below those observed in the normal group.

In the cortex, differences were less pronounced. For BDNF (Figure 1(b)), the only significant difference was an increase in expression in the fluoxetine treatment group compared to CUMS. Regarding GluN2B (Figure 1(f)), Riparin A restored its expression to control levels. No statistically significant differences were found for VEGF expression.

### 3.2. Cytokines Modulation by Riparin A

The effects of Riparin A on the levels of three proinflammatory and three anti‐inflammatory cytokines were analyzed in animals subjected to CUMS.

Regarding proinflammatory cytokines (Figure 2), Riparin A treatment significantly reduced TNF‐α and IL‐1β levels in both the hippocampus and cortex. However, it did not decrease IL‐6 levels; instead, IL‐6 expression was increased following treatment. Fluoxetine treatment also modulated cytokine levels, leading to significant reductions in TNF‐α and IL‐1β in both brain regions and in IL‐6 in hippocampus. Notably, Riparin A exhibited a superior effect compared to fluoxetine in reducing TNF‐α levels in both the hippocampus and cortex, as well as IL‐1β levels in the hippocampus.

Figure 2Effects of CUMS and treatments on proinflammatory cytokine levels (TNF‐α, IL‐1β, and IL‐6). The concentrations of TNF‐α in the hippocampus and cortex (a, b), IL‐1β (c, d), and IL‐6 (e, f) were measured in experimental groups using ELISA. ^∗^ *p* < 0.05, ^∗∗^ *p* < 0.01, ^∗∗∗^ *p* < 0.0005, and ^∗∗∗∗^ *p* < 0.0001.

**Figure:**
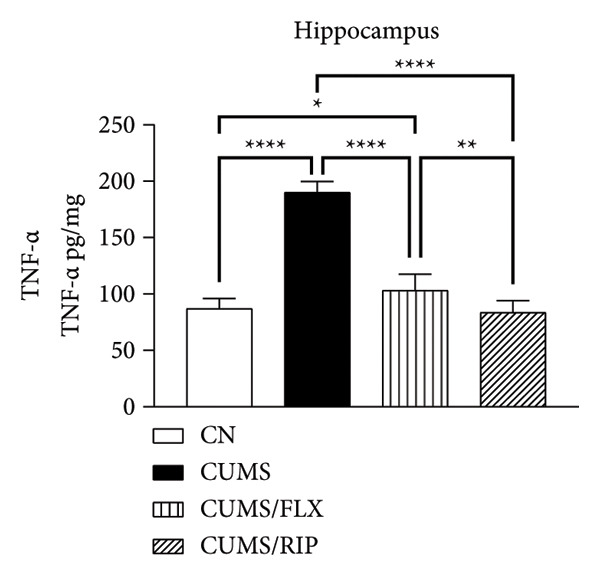
(a)

**Figure:**
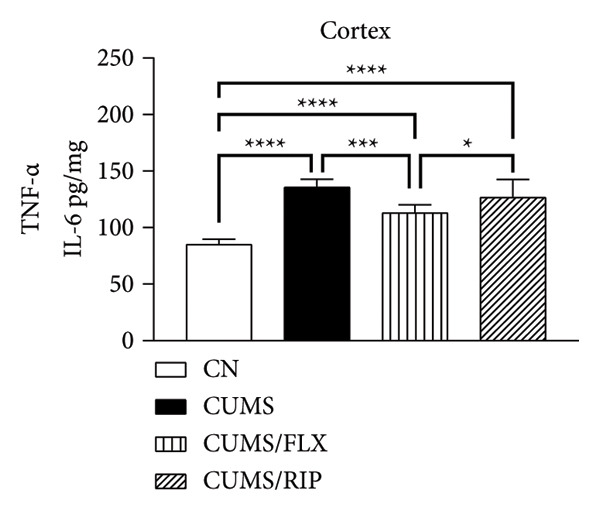
(b)

**Figure:**
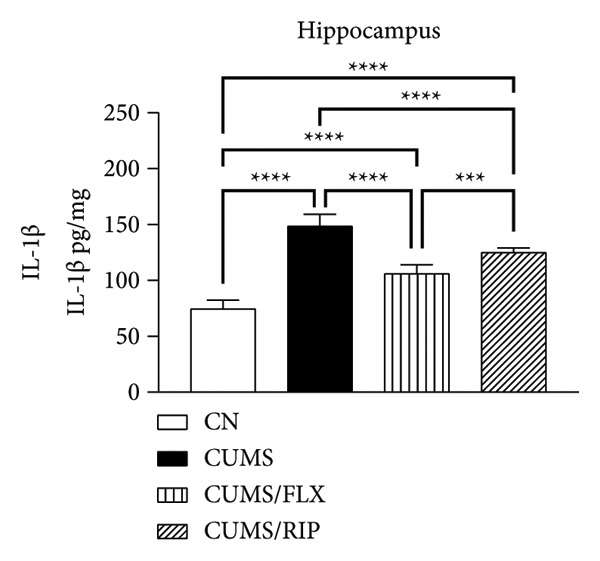
(c)

**Figure:**
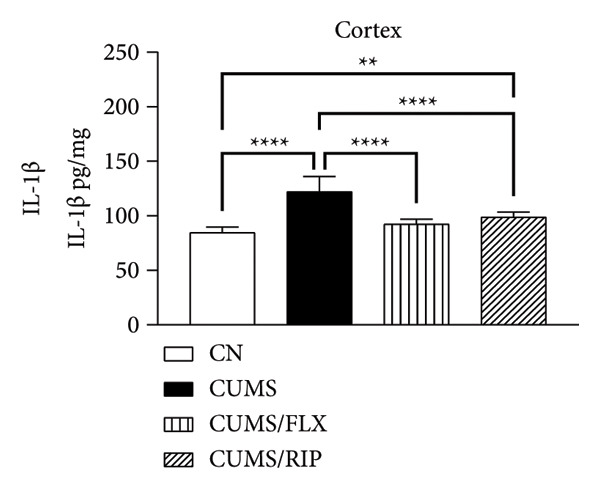
(d)

**Figure:**
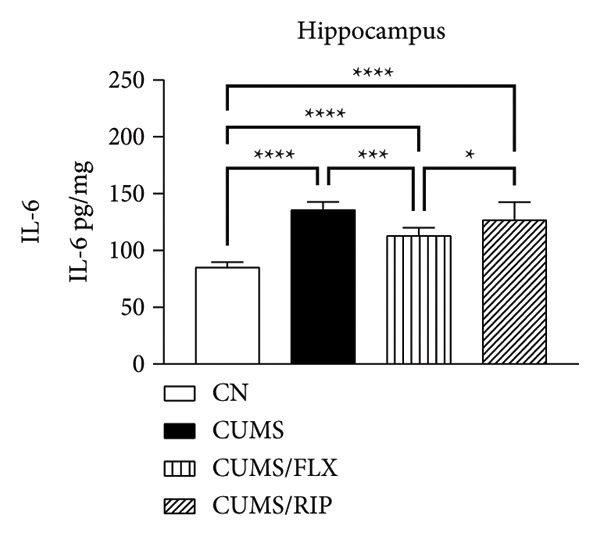
(e)

**Figure:**
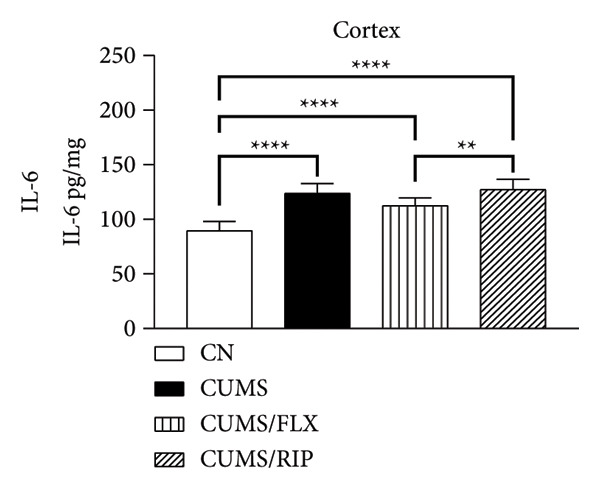
(f)

Riparin A treatment increased the expression of all analyzed cytokines compared to saline control, except for TNF‐α in the hippocampus.

In terms of anti‐inflammatory cytokines (Figure 3), both fluoxetine and Riparin A significantly increased IL‐10 expression in the hippocampus and cortex. Notably, Riparin A exhibited a stronger effect than fluoxetine in both brain regions.

Figure 3Effects of CUMS and treatments on anti‐inflammatory cytokine levels (IL‐4, IL‐10, and IL‐13). The concentrations of IL‐4 in the hippocampus and cortex (a, b), IL‐10 (c, d), and IL‐13 (e, f) were measured in experimental groups using ELISA. ^∗∗^ *p* < 0.01, ^∗∗∗^ *p* < 0.0005, and ^∗∗∗∗^ *p* < 0.0001.

**Figure:**
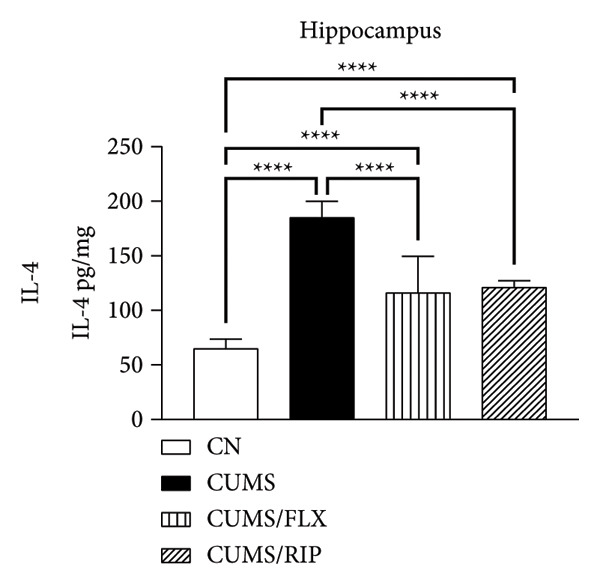
(a)

**Figure:**
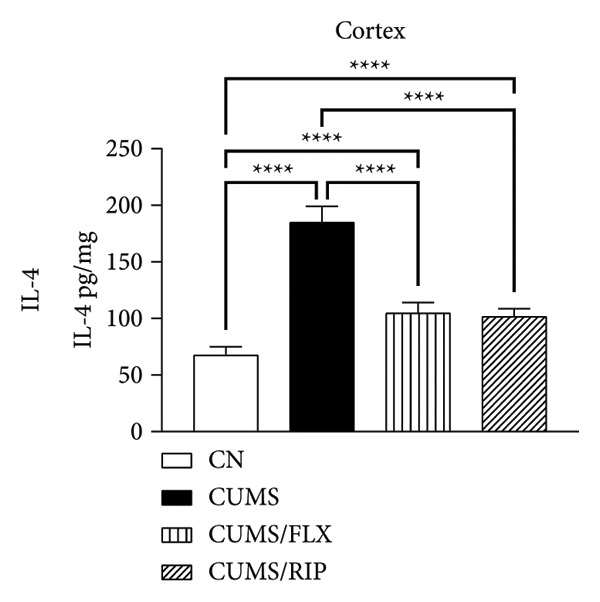
(b)

**Figure:**
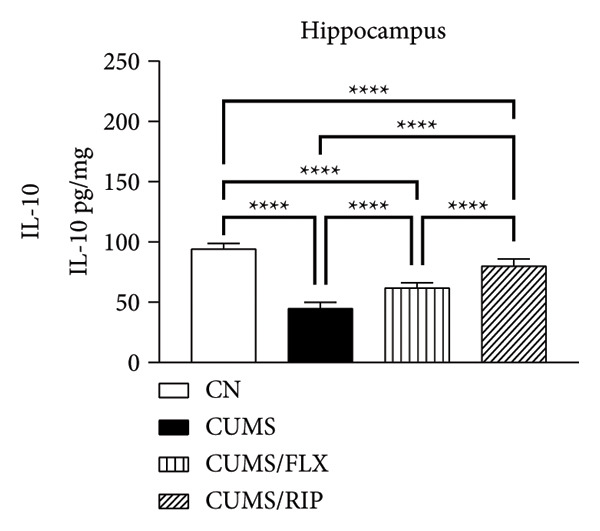
(c)

**Figure:**
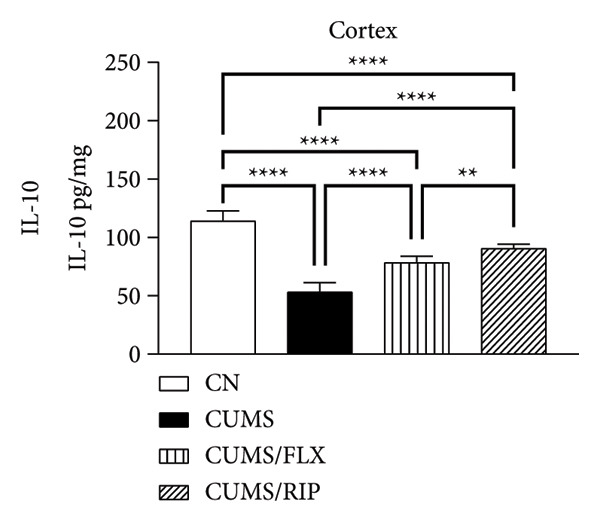
(d)

**Figure:**
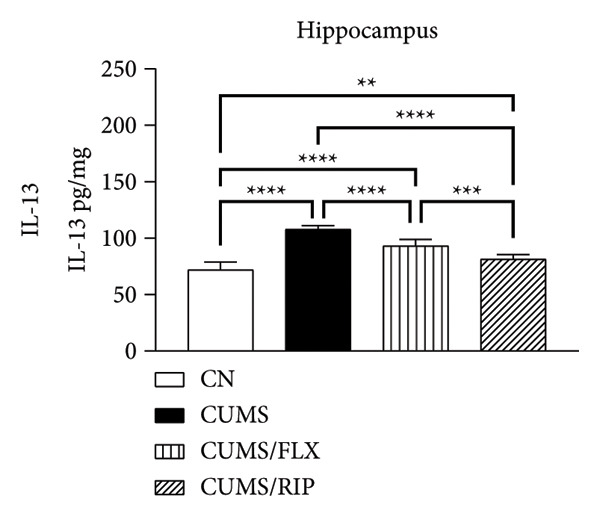
(e)

**Figure:**
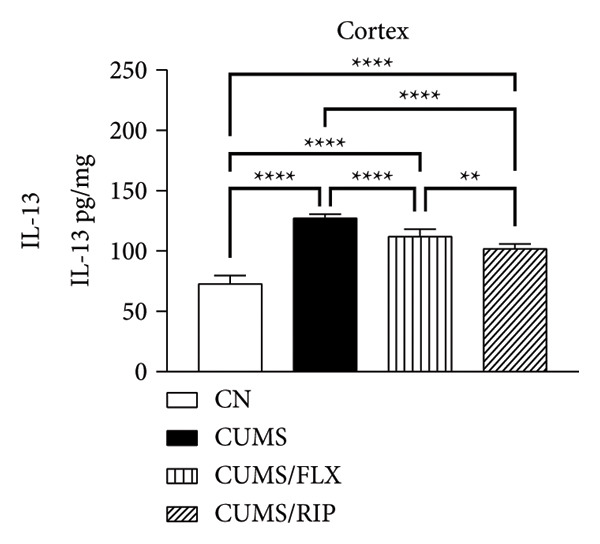
(f)

In contrast, IL‐4 and IL‐13 levels were reduced following both treatments in both brain areas. However, despite this reduction, their expression levels were higher than those observed in the saline control group.

## 4. Discussion

Riparin A is a synthetic analog of naturally occurring riparins, a class of compounds known for their antidepressant and anxiolytic properties. Similar to Riparins I, II, and III, which are predominantly found in medicinal plants of the genus Aniba, Riparin A has demonstrated promising neuroactive effects in preclinical studies [17–20].

While traditional antidepressants, such as selective serotonin reuptake inhibitors (SSRIs), primarily exert their effects by modulating monoaminergic neurotransmission, growing evidence suggests that neuroinflammatory and neurotrophic mechanisms also play a fundamental role in the pathophysiology of depression [29–32]. This has led to an increasing interest in novel pharmacological agents that target both neuroimmune and neurotrophic pathways, potentially offering greater therapeutic efficacy and fewer adverse effects compared to conventional treatments. In this context, the present study investigated the effects of Riparin A on neurotrophic gene expression and cytokine modulation in the hippocampus and cortex of animals subjected to the CUMS model of depression demonstrating that Riparin A exhibits neuroprotective and immunomodulatory properties, comparable to, and in some cases superior to, fluoxetine.

BDNF is one of the most widely distributed neurotrophins in the brain and plays a critical role in synaptic growth, development, and plasticity by modulating neuronal differentiation [33, 34]. Consistent with previous studies, where BDNF gene expression levels are markedly downregulated in the hippocampus and medial prefrontal cortex of individuals with depression [35], and VEGF is similarly reduced in the hippocampus of animals exposed to depressive‐like conditions [26], our results demonstrated that CUMS significantly decreased BDNF and VEGF expression in the hippocampus. Conversely, CUMS increased GluN2B expression, a finding that aligns with the studies reporting that antagonism of GluN2B‐containing NMDA receptors induces antidepressant effects causing significant behavioral changes in both clinical patients and rodent models of depression [36–40]. These results reinforce the involvement of glutamatergic dysregulation in depression and suggest that Riparin A’s antidepressant effects may, at least in part, be mediated by its ability to facilitate synaptic plasticity and remodeling, thereby alleviating depressive symptoms [35, 41].

Both Riparin A and fluoxetine treatments counteracted these effects, restoring BDNF and VEGF expression while reducing GluN2B expression. However, while fluoxetine partially increased BDNF levels, Riparin A fully restored hippocampal BDNF expression to control levels, suggesting a stronger effect on neurotrophin regulation.

VEGF expression in the hippocampus did not differ significantly between control and CUMS groups, indicating that its dysregulation may be less pronounced than that of BDNF under chronic stress conditions. Nevertheless, fluoxetine increased VEGF expression following stress, while Riparin A elevated VEGF levels beyond control values, reinforcing its potential role in promoting neurovascular support since it contributes significantly to neuronal survival, maturation, and synaptic plasticity, with direct involvement in neurogenesis, processes that have been associated with improved behavioral effects in depression models [42, 43].

In the cortex, Riparin A did not induce significant alterations in BDNF or VEGF expression, with fluoxetine being the only treatment that significantly increased BDNF levels in this brain region. However, Riparin A restored cortical GluN2B expression to baseline, an effect not observed with fluoxetine. Despite these specific effects, the overall expression levels of these neurotrophic genes in the cortex remained unchanged, indicating that Riparin A’s primary neuroprotective effects may be more pronounced in the hippocampus.

Regarding inflammation, the inflammatory hypothesis of depression proposes that proinflammatory cytokines contribute to neuroinflammation, neuronal dysfunction, and behavioral impairments, playing a pivotal role in the pathophysiology of the disorder [3, 15]. Elevated levels of proinflammatory cytokines have been detected in key brain regions involved in mood regulation, cognition, and emotional processing, including the hippocampus, prefrontal cortex, anterior cingulate cortex, and amygdala [44–46]. These cytokines can disrupt neuronal communication, alter neurotransmitter dynamics, and impair neurogenesis, leading to dysregulated mood and cognitive deficits [47–50]. Some studies have demonstrated that the administration of IL‐10, IL‐13, and IL‐1 leads to improvement and recovery of depressive symptoms in animal models [51–53]. Moreover, inflammation‐driven modulation of key neurotrophic factors such as BDNF, VEGF, and GluN2B has been recognized as an essential component of depression pathophysiology and a crucial target for effective treatment [3, 4].

Here, Riparin A exerted an anti‐inflammatory effect by reducing hippocampal and cortical levels of TNF‐α and IL‐1β, two key proinflammatory cytokines associated with depressive‐like behaviors [54]. Interestingly, IL‐6 levels were increased following Riparin A treatment, a response also observed with fluoxetine but only in the cortex. While IL‐6 is typically considered proinflammatory, it has context‐dependent effects and can exhibit neuroprotective roles under certain conditions [55, 56].

In terms of anti‐inflammatory cytokines, we found that both fluoxetine and Riparin A significantly increased IL‐10 levels in both brain regions, with Riparin A exerting a stronger effect than fluoxetine. Given that IL‐10 plays a crucial role in counteracting neuroinflammation and promoting resilience to stress [14], this result highlights Riparin A’s potential as a potent anti‐inflammatory agent. In contrast, IL‐4 and IL‐13 were downregulated by both treatments, although their levels remained higher than those of saline controls, suggesting a partial preservation of their regulatory effects.

Despite the relevant molecular findings presented, several limitations should be acknowledged. First, although the expression of BDNF was evaluated in specific brain regions, its precursor form, proBDNF, and the associated receptors TrkB and phosphorylated TrkB (p‐TrkB) were not measured. This limits a more comprehensive understanding of the neurotrophic signaling pathways and their activation status.

Another important limitation is the lack of behavioral assessments. Although the CUMS model was employed, no behavioral tests were performed to confirm the induction of depressive‐like behavior or the antidepressant efficacy of Riparin A at the behavioral level. Including such tests would have strengthened the functional interpretation of the molecular alterations observed.

Additionally, the exclusive use of male rats was chosen to minimize the biological variability associated with the estrous cycle in females. Our primary objective was to establish a baseline understanding of the molecular effects under controlled conditions before extending the investigation to both sexes. We fully acknowledge the critical importance of sex as a biological variable, particularly in neuropsychiatric research, and future studies are planned to include female subjects to provide a more comprehensive and translational understanding of the findings.

In summary, our results demonstrate that Riparin A modulates key neurotrophic factors and cytokines associated with depression, exerting effects comparable to or greater than fluoxetine in certain contexts. Its ability to fully restore BDNF levels, regulate VEGF, suppress TNF‐α, and enhance IL‐10 expression highlights its potential as a novel antidepressant candidate. These findings support further investigations into Riparin A’s mechanisms and its potential therapeutic application in depressive disorders. Further studies are warranted to elucidate the precise mechanisms underlying these effects and their potential clinical implications. Comprehensive pharmacological and biochemical characterization of various Riparins is essential to gain insights into their structure–activity relationships, thus broadening our understanding of these compounds. These results suggest medical and pharmaceutical significance, underscoring the potential biotechnological interest in the pharmacological group of Riparins.

## Ethics Statement

The study was conducted in accordance with the Declaration of Helsinki and approved by the Institutional Review Board (or Ethics Committee) of the University of Ribeirão Preto—UNAERP (protocol code: 10/2015, date of approval: 4.10.2015).

## Consent

The authors have nothing to report.

## Conflicts of Interest

The authors declare no conflicts of interest.

## Funding

This study was financed by the Coordination for the Improvement of Higher Education Personnel (Coordenação de Aperfeiçoamento Pessoal de Nível Superior—Brazil—CAPES)‐Finance Code 001 and the National Council for Scientific and Technological Development (Conselho Nacional de Desenvolvimento Científico e Tecnologico—CNPq).

## Data Availability

The data that support the findings of this study are available from the corresponding author upon reasonable request.
